# Longitudinal Dynamics of a Blood Transcriptomic Signature of Tuberculosis

**DOI:** 10.1164/rccm.202103-0548OC

**Published:** 2021-03-30

**Authors:** Humphrey Mulenga, Munyaradzi Musvosvi, Simon C. Mendelsohn, Adam Penn-Nicholson, Stanley Kimbung Mbandi, Andrew Fiore-Gartland, Michèle Tameris, Simbarashe Mabwe, Hadn Africa, Nicole Bilek, Fazlin Kafaar, Shabaana A. Khader, Balie Carstens, Katie Hadley, Chris Hikuam, Mzwandile Erasmus, Lungisa Jaxa, Rodney Raphela, Onke Nombida, Masooda Kaskar, Mark P. Nicol, Slindile Mbhele, Judi Van Heerden, Craig Innes, William Brumskine, Andriëtte Hiemstra, Stephanus T. Malherbe, Razia Hassan-Moosa, Gerhard Walzl, Kogieleum Naidoo, Gavin Churchyard, Mark Hatherill, Thomas J. Scriba, the CORTIS Study Team

**Affiliations:** ^1^ South African Tuberculosis Vaccine Initiative, Institute of Infectious Disease and Molecular Medicine and Division of Immunology, and; ^4^ Division of Medical Microbiology, Department of Pathology, University of Cape Town, Cape Town, South Africa;; ^2^ Vaccine and Infectious Disease Division, Fred Hutchinson Cancer Research Center, Seattle, Washington;; ^3^ Department of Molecular Microbiology, Washington University in St. Louis, St. Louis, Missouri;; ^5^ Division of Infection and Immunity, School of Biomedical Sciences, University of Western Australia, Perth, Australia;; ^6^ The Aurum Institute, Johannesburg, Guateng, South Africa;; ^7^ Department of Science and Technology/National Research Foundation Centre of Excellence for Biomedical TB Research and SAMRC Centre for Tuberculosis Research, Division of Molecular Biology and Human Genetics, Department of Biomedical Sciences, Faculty of Medicine and Health Sciences, Stellenbosch University, Cape Town, South Africa;; ^8^ Centre for the AIDS Programme of Research in South Africa (CAPRISA), Durban, South Africa;; ^9^ Medical Research Council-CAPRISA HIV-Tuberculosis Pathogenesis and Treatment Research Unit, Doris Duke Medical Research Institute, University of KwaZulu-Natal, Durban, South Africa; and; ^10^ School of Public Health, University of Witwatersrand, Johannesburg, South Africa

**Keywords:** mRNA, biomarkers, HIV, respiratory tract infections, *Mycobacterium tuberculosis*

## Abstract

**Rationale:**

Performance of blood transcriptomic tuberculosis (TB) signatures in longitudinal studies and effects of TB-preventive therapy and coinfection with HIV or respiratory organisms on transcriptomic signatures has not been systematically studied.

**Objectives:**

We evaluated longitudinal kinetics of an 11-gene blood transcriptomic TB signature, RISK11, and effects of TB-preventive therapy (TPT) and respiratory organisms on RISK11 signature score, in HIV-uninfected and HIV-infected individuals.

**Methods:**

RISK11 was measured in a longitudinal study of RISK11-guided TPT in HIV-uninfected adults, a cross-sectional respiratory organisms cohort, or a longitudinal study in people living with HIV (PLHIV). HIV-uninfected RISK11^+^ participants were randomized to TPT or no TPT; RISK11^−^ participants received no TPT. PLHIV received standard-of-care antiretroviral therapy and TPT. In the cross-sectional respiratory organisms cohort, viruses and bacteria in nasopharyngeal and oropharyngeal swabs were quantified by real-time quantitative PCR.

**Measurements and Main Results:**

RISK11^+^ status was transient in most of the 128 HIV-negative participants with longitudinal samples; more than 70% of RISK11^+^ participants reverted to RISK11^−^ by 3 months, irrespective of TPT. By comparison, reversion from a RISK11^+^ state was less common in 645 PLHIV (42.1%). Non-HIV viral and nontuberculous bacterial organisms were detected in 7.2% and 38.9% of the 1,000 respiratory organisms cohort participants, respectively, and among those investigated for TB, 3.8% had prevalent disease. Median RISK11 scores (%) were higher in participants with viral organisms alone (46.7%), viral and bacterial organisms (42.8%), or prevalent TB (85.7%) than those with bacterial organisms other than TB (13.4%) or no organisms (14.2%). RISK11 could not discriminate between prevalent TB and viral organisms.

**Conclusions:**

Positive RISK11 signature status is often transient, possibly due to intercurrent viral infection, highlighting potentially important challenges for implementation of these biomarkers as new tools for TB control.

**At a Glance Commentary**

**Scientific Knowledge on the Subject**

Improvement in strategies for active tuberculosis (TB) case finding requires sensitive, nonsputum-based screening tests that can identify individuals with undiagnosed TB or those at highest risk of disease progression. Multiple host-blood gene expression (transcriptomic) signatures that show promising diagnostic and/or prognostic performance in case-control studies have been developed. However, performance of such signatures in longitudinal studies and effects of TB-preventive therapy and coinfection with HIV or respiratory organisms on transcriptomic signatures has not been systematically studied.

**What This Study Adds to the Field**

We present evidence from human cohort studies that positive status for the RISK11 blood transcriptomic signature of TB was unexpectedly transient, both in individuals who received TB-preventive therapy and in untreated individuals. Individuals with viruses detected by respiratory sampling, or with HIV infection, had elevated RISK11 signature scores, highlighting viral infections as important confounding factors for transcriptomic TB signatures.

Up to 1.7 billion people are estimated to be infected with *Mycobacterium tuberculosis* (*Mtb*) (1); however, less than 10% may progress to tuberculosis (TB) disease in their lifetime (2, 3). Diagnosis and treatment currently depend on screening for symptoms consistent with TB and collection of sputum for microbiological testing. However, prevalence of subclinical (asymptomatic) TB is high, such that symptom-based case-finding approaches would fail to detect approximately 50% of cases (4).

Biomarkers that allow identification of individuals with both subclinical and clinical disease are urgently needed. Such tests may be useful to guide confirmatory testing, or for screen-and-treat strategies to direct short-course TB-preventive therapy (TPT) to persons at highest risk of progression to TB. Many blood transcriptomic TB signatures with promise as TB triage tests (5), and as tests for predicting progression to TB disease (6), have been reported (7–9).

We developed a PCR-based 11-gene signature (RISK11) that predicted development of TB disease in individuals with *Mtb* infection up to 12 months preceding TB diagnosis and had promising diagnostic performance for TB (10–12). Diagnostic performance of RISK11 for TB in a prospective study of symptomatic HIV-uninfected adults in a TB endemic setting was very promising (13). Prognostic performance for short-term prediction of incident TB was also good, but RISK11-guided TPT with 3 months of weekly rifapentine and isoniazid (3HP) did not reduce progression to TB disease over 15 months (13).

Although treatment of TB disease has been shown to reduce RISK11 score (12, 14), the effect of TPT on transcriptomic signature scores in individuals without TB disease is not known. Longitudinal dynamics of RISK11 or other transcriptomic signatures in healthy persons without TPT have also not been described. If transcriptomic signatures are to be implemented for serial TB screening, an understanding of biomarker dynamics in the face of changing host, pathogen, and treatment factors is needed.

One factor that might affect transcriptomic signature score is viral infection. Among participants without TB disease, detectable HIV plasma viral load is known to be associated with elevated signature scores compared with undetectable viral load, likely due to induction of type I IFN and elevated expression of IFN-stimulated genes (ISGs), which comprise RISK11 (12). Another ISG-inducing viral infection, influenza (15), has also been shown to affect transcriptomic signature scores. However, the effect of other common upper respiratory infections on TB signatures remains unexplored.

We evaluated RISK11 dynamics in adults with and without HIV, assessed the effect of TPT on signature dynamics, and evaluated the effect of upper respiratory tract organisms on RISK11 score. Some of the results of these studies have been previously reported in the form of an abstract (16). Deidentified RISK11 signature scores, probe-level PCR data, TB endpoint data, and upper respiratory tract organism data from all participants are deposited in Zivahub (https://doi.org/10.25375/uct.14610582.v1), an open access data repository hosted by the University of Cape Town’s data repository powered by Figshare for Institutions.

## Methods

### Study Design and Study Populations

**Figure 1.**
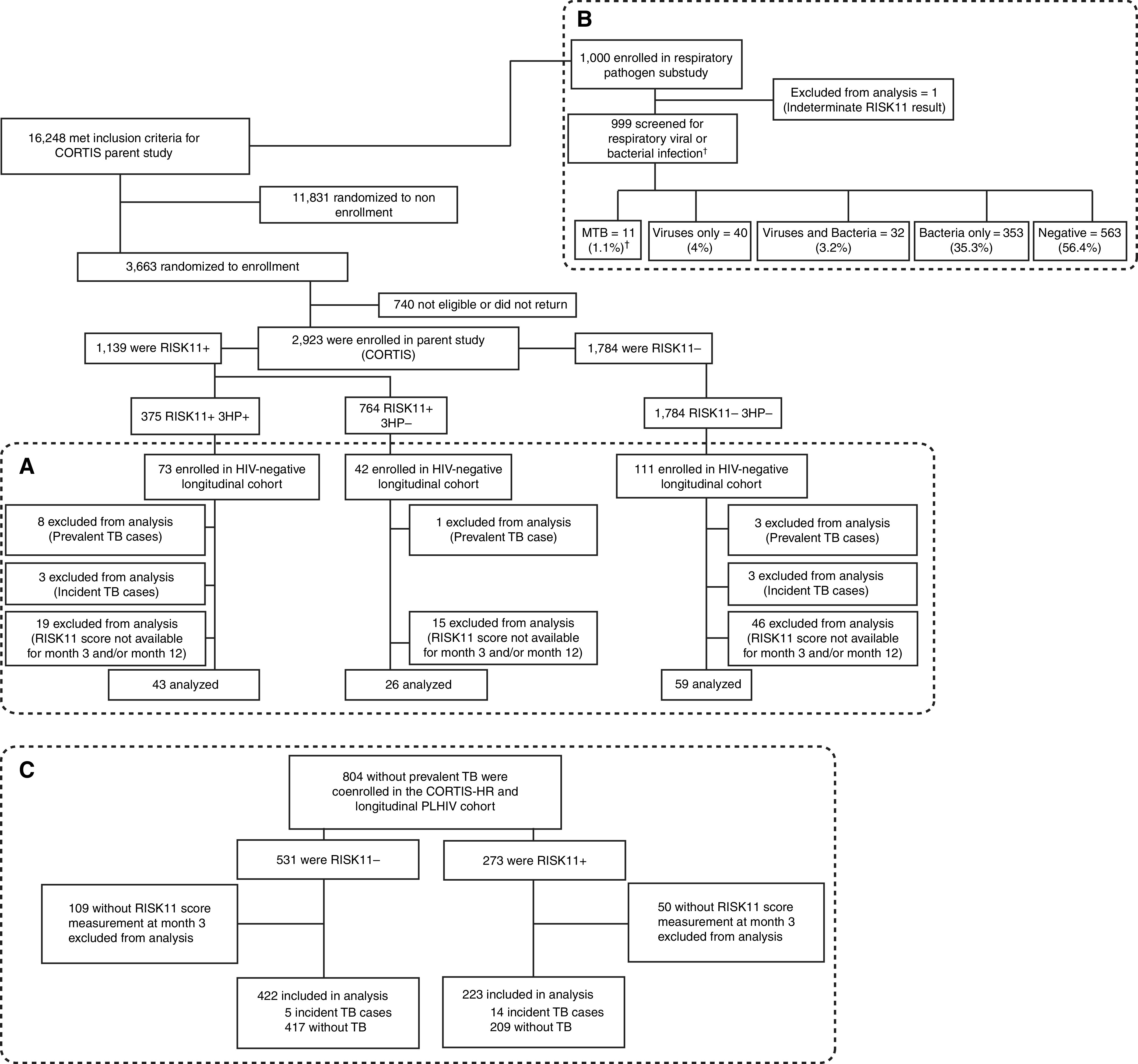
Study flow diagram. HIV-uninfected participants were recruited from the CORTIS (Correlate of Risk Targeted Intervention Study) parent study and coenrolled into the (*A*) HIV-uninfected longitudinal cohort and (*B*) respiratory organisms cohort. All participants living with HIV (PLHIV) enrolled in the CORTIS-HR (Validation of Correlates of Risk of TB Disease in High Risk Populations) parent study with a baseline RISK11 score and without prevalent tuberculosis (TB) were eligible for inclusion in the (*C*) PLHIV longitudinal cohort.^ †^Out of 999 participants enrolled in the respiratory organisms cohort, only 286 (28.6%) participants were coenrolled in the CORTIS parent study and investigated for TB at enrollment (Figure E1); 11/286 (3.8%) had prevalent TB confirmed by MTB liquid culture and/or Xpert MTB/RIF (Cepheid). 3HP = 3 months’ rifapentine and isoniazid preventive therapy; MTB = *Mycobacterium tuberculosis.*

We enrolled HIV-uninfected volunteers into two observational cohorts nested in the CORTIS (Correlate of Risk Targeted Intervention Study) trial (13) (ClinicalTrials.gov: NCT02735590) and HIV-infected volunteers into a cohort nested in the observational CORTIS-HR (Validation of Correlates of Risk of TB Disease in High Risk Populations) study (17), respectively (Figure 1; Figure E1 in the online supplement). These three cohorts allowed evaluation of *1*) transcriptomic signature dynamics in HIV-uninfected and HIV-infected individuals; *2*) transcriptomic signature dynamics with and without TPT; and *3*) effects of upper respiratory organisms on the transcriptomic signature.

#### Parent studies

Briefly, healthy adult (18–60 yr) community volunteers with and without HIV residing in TB endemic communities in South Africa were recruited. Eligible participants did not have known TB disease, or household exposure to a person with multi-drug-resistant TB, within the last 3 years. Participants were investigated for TB at enrollment, end of study, and upon detection of symptoms consistent with TB during study follow-up of 15 months. Participants were tested with the RISK11 biomarker at screening and stratified as RISK11^+^ or RISK11^−^ based on an *a priori* test threshold of 60% (13). HIV-uninfected RISK11^+^ participants in the CORTIS trial were randomized to receive TPT (weekly high-dose isoniazid and rifapentine for 12 weeks [3HP]) or no intervention; HIV-uninfected RISK11^−^ participants did not receive TPT. People living with HIV (PLHIV) were referred for standard-of-care antiretroviral therapy (ART) and TPT. Study protocols were approved by the institutional human research ethics committees of participating sites. The CORTIS trial was approved by the South African Health Products Regulatory Authority. All participants provided written, informed consent for participation.

#### Longitudinal cohorts

In the HIV-uninfected longitudinal cohort, we aimed to determine the dynamics of the RISK11 signature over 12 months of follow-up, and the effect of 3HP on RISK11 signature dynamics, by repeat measurement of RISK11 at 3 and 12 months. RISK11^+^ participants in the 3HP^+^ treatment arm, and both RISK11^+^ and RISK11^−^ participants in the 3HP^−^ arm, were enrolled at two sites (Worcester and Ravensmead).

In the HIV-infected longitudinal cohort, we enrolled RISK11^+^ and RISK11^−^ participants at five sites (Worcester, Ravensmead, Durban, Klerksdorp, and Rustenberg) and measured RISK11 at enrollment and Month 3 to determine RISK11 dynamics through 3 months.

Participants with prevalent TB at enrollment and those without RISK11 measurement at Month 3 or Month 12 (HIV-uninfected longitudinal cohort) were excluded from analysis of signature dynamics. TB screening and RISK11 assay were performed as described (13). Details are in the online supplement.

#### Respiratory organisms cohort

A subset of HIV-uninfected participants screened for CORTIS were contemporaneously and consecutively enrolled (Figure E1), irrespective of symptoms or signs of upper respiratory tract infections, at one site (Worcester). Paired nasopharyngeal and oropharyngeal flocked swabs (FLOQSwabs; COPAN Diagnostics Inc.) were collected and stored in 1.5 ml of Primestore buffer (Longhorn Vaccines and Diagnostics) at −80°C. Participants coenrolled into the CORTIS trial were additionally investigated for TB disease at baseline; those only enrolled into the respiratory organisms cohort were not investigated for TB (Figure E1).

### Respiratory Pathogen Assay

For detection of respiratory viral and bacterial organisms, nucleic acid was extracted from swab samples using the Qiasymphony Virus/Bacteria Mini Kit (Qiagen) and quantified using a multiplex real-time quantitative PCR assay kit (Fast Track Diagnostics Respiratory Pathogens 33 Kit), according to the manufacturer’s instructions, on the CFX96 Touch System lightcycler platform (Bio-Rad). This kit is designed to detect a panel of respiratory tract bacteria, viruses, and fungi (Table E4).

### Statistical Analysis

Statistical analyses were performed in RStudio version 1.0.153 (RStudio PBC) or Stata version 16 (StataCorp). Receiver operating characteristic area under the curve (AUC) was computed to estimate diagnostic or prognostic performance of RISK11 measured at enrollment versus Month 3 through 15 months’ follow-up and to determine whether RISK11 can differentiate participants with or without organisms. The 95% confidence interval (CI) for AUC and differences in paired AUC were estimated using a percentile bootstrap with 10,000 iterations. A Wilcoxon rank-sum test was used to test for differences in RISK11 scores between participants with and without respiratory organisms. Participants with viral and bacterial upper respiratory tract codetection were included in the viral organism group, as they had similar RISK11 score distributions.

A multivariable linear regression model was used to estimate the effect of detection of upper respiratory organisms on RISK11 score. A logit transformation was performed on RISK11 score before fitting the model, as RISK11 score is a proportion. The multivariable model was built using the likelihood ratio test method. First, an initial model with just RISK11 was fitted. Next, nested models were fitted and compared with the initial model. The variable in the model with the smallest Akaike information criterion value and making the most significant contribution was then added to the initial model. The process was repeated until no variable made a significant contribution to the model. Analyses in the 286 participants from the respiratory organisms cohort who were coenrolled in CORTIS were adjusted with sampling weights to reflect the screened population (13). Enrollment into CORTIS was based on RISK11 status. For reasons of trial efficiency, approximately 79% of all eligible RISK11^+^ and only 13% of all eligible RISK11^−^ participants were enrolled. Because of this enrichment in RISK11^+^ participants in the enrolled population, sampling weights of 1.263 and 7.920 were assigned to RISK11^+^ and RISK11^−^ individuals, respectively, to obtain estimates of the screened population. An α of less than 0.05 was considered statistically significant in all analyses. All analyses in this substudy were performed *post hoc* without a prespecified statistical analysis plan.

## Results

### RISK11 Reversion Is Common, Irrespective of TB-Preventive Therapy

**Figure 2.**
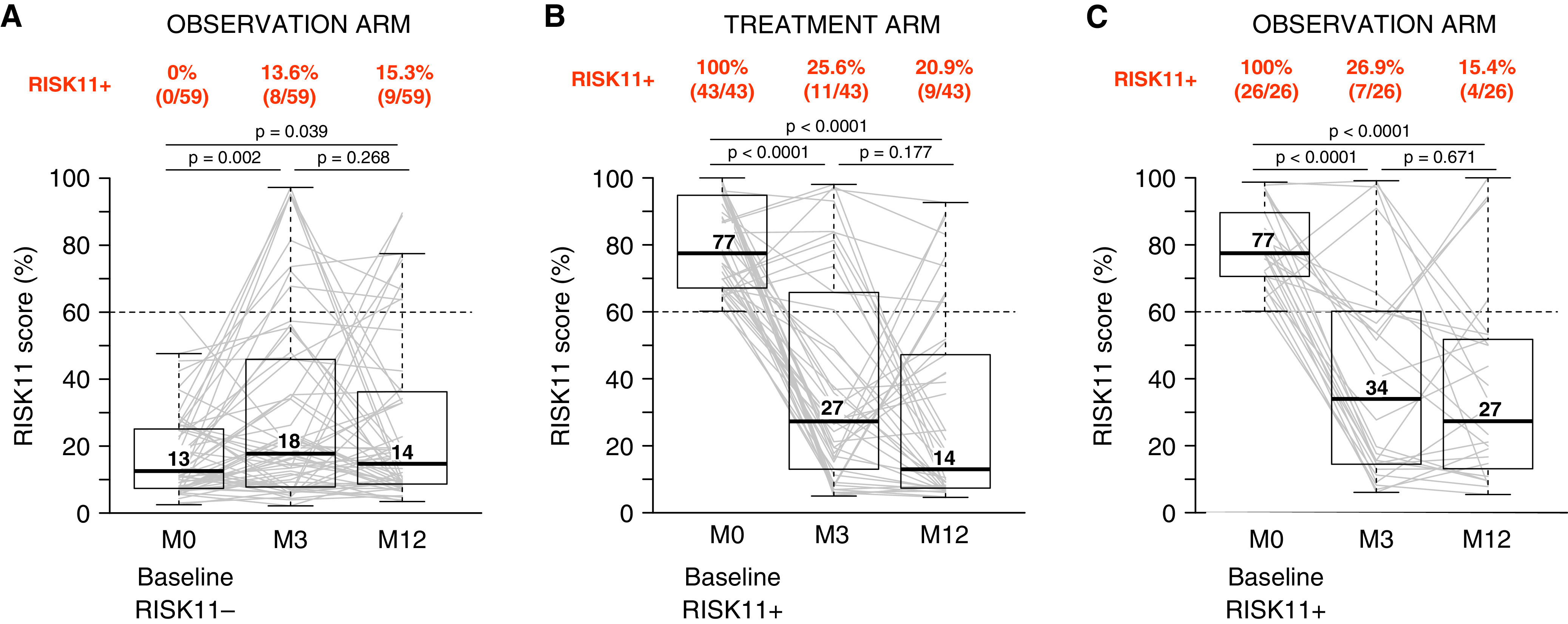
RISK11 positivity is transient among most adults in the HIV-uninfected longitudinal cohort. RISK11 scores at enrollment (M0), and 3 months (M3) and 12 months (M12) after enrollment in (*A*) baseline RISK11^−^ participants in the observational arm (*n* = 59), (*B*) baseline RISK11^+^ participants in the 3 months’ rifapentine and isoniazid preventive therapy–positive treatment arm (*n* = 43), and (*C*) baseline RISK11^+^ participants in the observational arm (*n* = 26). All prevalent and incident cases were excluded. Boxes represent the interquartile range, and horizontal lines represent medians. The whiskers represent the lowest and highest RISK11^+^ score within 1.5 times the interquartile range from the lower quantile and upper quantile, respectively. The proportions of RISK11^+^ participants at each time point are indicated above each plot. The Wilcoxon signed-rank test was used to compare RISK11 scores between time points.

In total, 226 participants were enrolled into the HIV-uninfected longitudinal cohort. Data were available at all three time points for 128 participants without TB disease (43 RISK11^+^/3HP^+^; 26 RISK11^+^/3HP^−^; and 59 RISK11^−^/3HP^−^) (Figure 1A and Table E1). Few RISK11^−^ participants at screening converted to RISK11^+^ at Month 3 (13.6%, 8/59) and/or Month 12 (15.3%, 9/59) (Figure 2A).

Among RISK11^+^ participants at screening who received 3HP, most reverted to RISK11^−^ by Month 3 (74.4%, 32/43) or Month 12 (79.1%, 34/43) (Figure 2B). Surprisingly, we observed the same phenomenon for RISK11^+^ participants who did not receive 3HP, most of whom reverted to RISK11^−^ by Month 3 (73.1%, 19/26) or Month 12 (84.6%, 22/26) (Figure 2C). Only 4 of 71 RISK11^+^ participants (5.6%) remained persistently RISK11^+^ through the 3- and 12-month visits, none of whom developed incident TB. Together these results suggest that RISK11 positivity is transient in most people without HIV infection and that 3HP, which is thought to clear *Mtb* infection, did not increase the RISK11 reversion rate above that observed in 3HP-untreated individuals. Prevalence of IFN-γ release assay-positive individuals were not different between the groups (Table E1).

### RISK11 Reversion Is Less Common in PLHIV, but ART Initiation Leads to RISK11 Reversion

**Figure 3.**
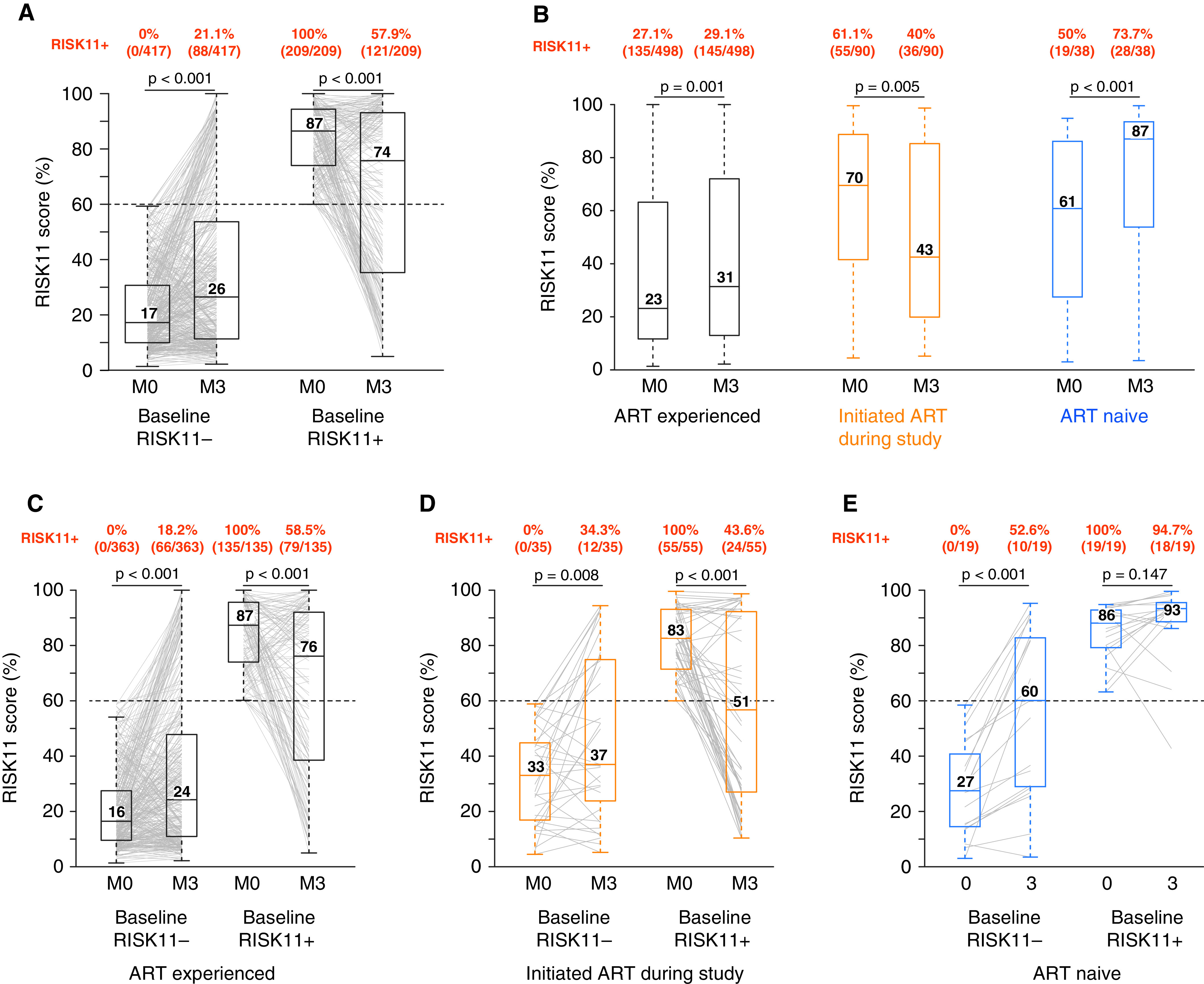
RISK11 reversion is less common among participants of the people living with HIV (PLHIV) longitudinal cohort. (*A*) RISK11 scores at enrollment (M0) and 3 months (M3) for all participants in the PLHIV longitudinal cohort, excluding all prevalent and incident cases. Participants have been stratified by baseline RISK11 status, and the proportions of RISK11-positive (RISK11^+^) participants at each time point are indicated above each plot. (*B*) RISK11 scores at enrollment (M0) and 3 months (M3) for all participants in the PLHIV longitudinal cohort, excluding all prevalent and incident tuberculosis (TB) cases, stratified by antiretroviral therapy (ART) status. The proportions of RISK11^+^ participants at each time point are indicated above each plot. (*C–E*) RISK11 scores at M0 and M3 for ART-experienced participants (*C*), participants who initiated ART after enrollment (*D*), and ART-naive participants (*E*), excluding all prevalent and incident TB cases. Boxes represent the interquartile range, and horizontal lines represent medians. The whiskers represent the lowest and highest RISK11^+^ score within 1.5 times the interquartile range from the lower quantile and upper quantile, respectively. Participants have been stratified by baseline RISK11 status, and the proportions of RISK11^+^ participants at each time point are indicated above each plot. The Wilcoxon signed-rank test was used to compare RISK11 scores between time points.

Next, we evaluated the dynamics of RISK11 in the longitudinal HIV-infected cohort. RISK11 data were available at both enrollment and Month 3 for 645 participants without prevalent TB (422 RISK11^−^ and 223 RISK11^+ ^at enrollment) (Figure 1C and Table E2). Nineteen of 645 participants progressed to incident TB. Among the 626 participants (417 RISK11^−^ and 209 RISK11^+^ ) without incident TB, 21.1% (88/417) of those who were RISK11^−^ at enrollment converted to RISK11^+^ at 3 months after enrollment, a conversion rate similar to that observed in the HIV-uninfected cohort (13.6%, Fisher’s exact test, *P* = 0.2) (Figure 3A). However, the percentage of PLHIV without incident TB who were RISK11^+^ at enrollment and reverted to RISK11^−^ by Month 3 (42.1%, 88/209) (Figure 3A) was significantly lower than in the HIV-uninfected cohort (73.1%, *P* = 0.022) (Figure 2C), suggesting that HIV infection may contribute to persistence of RISK11^+^ status.

We then stratified PLHIV by isoniazid preventive therapy (IPT) status to determine the effect of IPT on the longitudinal RISK11 score. RISK11 conversion and reversion rates observed between enrollment and Month 3 were similar among the following subgroups: 38 participants on IPT at baseline (conversion 19.4%, 6/31, and reversion 42.9%, 3/7) (Figure E2A), 174 participants who initiated IPT through Month 3 (conversion 20.3%, 26/128, and reversion 45.7%, 21/46) (Figure E2B), and 414 participants who did not take IPT during this period (conversion 21.7%, 56/258, and reversion 41.0%, 64/156) (Figure E2C). These results in PLHIV support our observation in the HIV-uninfected cohort that TPT did not affect the RISK11 reversion rate.

When stratified by ART status, enrollment RISK11 scores were lower in ART-experienced (median [IQR], 23.2% [11.7–63.2%]) than ART-naive participants (median [IQR], 66.7% [35.9–87.1%]; *P* < 0.0001). Duration of ART, CD4 count, detectable plasma viral load are in Table E2. Among 498 ART-experienced participants, the proportion that were RISK11^+ ^remained constant through Month 3 (27.1%, *n* = 135 RISK11^+^ at enrollment vs. 29.1%, *n* = 145 at Month 3; *P* = 0.53) (Figure 3B). However, among 90 ART-naive participants who initiated ART during the study, median scores declined from 69.5% (IQR, 41.7–88.2%) to 42.5% (IQR, 20.1–84.3%; *P* = 0.0046) (Figure 3B). Among RISK11^+^ participants who were ART-experienced or initiated ART during the study, 41.5% (56/135) and 56.4% (31/55) had reverted to RISK11^−^ by Month 3, respectively (Figures 3C and 3D). By contrast, among 38 participants who remained ART-naive through Month 3, median RISK11 scores increased from 60.8% (IQR, 27.6–85.8%) at enrollment to 87.0% (IQR, 55.4–93.4%) through Month 3 (*P* < 0.0001) (Figure 3B), with only 5.3% (1/19) reverting to RISK11^−^ and 52.6% (10/19) converting to RISK11^+^ (Figure 3E). These findings support prior evidence that HIV viraemia upregulates ISG expression.

Among 19 participants (5 RISK11^−^ and 14 RISK11^+^) in the HIV-infected cohort who progressed to incident TB through median 13.4 (IQR, 10.9–15) months’ follow-up, median RISK11 scores increased from 73.2% (IQR, 58.2–89.6%) at enrollment to 88.1% (IQR, 56.8–95.7%) by Month 3 (*P* = 0.59) (Figure E3A). The proportion of RISK11^+^ participants at enrollment versus Month 3 was not different (*P* = 1). RISK11 prognostic performance at enrollment (AUC, 73.1%; 95% CI, 62.6–82.1%) or at Month 3 (AUC, 73.5%; 95% CI, 62.0–83.8%) for microbiologically confirmed incident TB through 15 or 12 months’ follow-up, respectively, was not different (*P* = 0.93) (Figure E3B).

### Viral Upper Respiratory Tract Organisms Are Associated with Elevated RISK11 Scores

**Figure 4.**
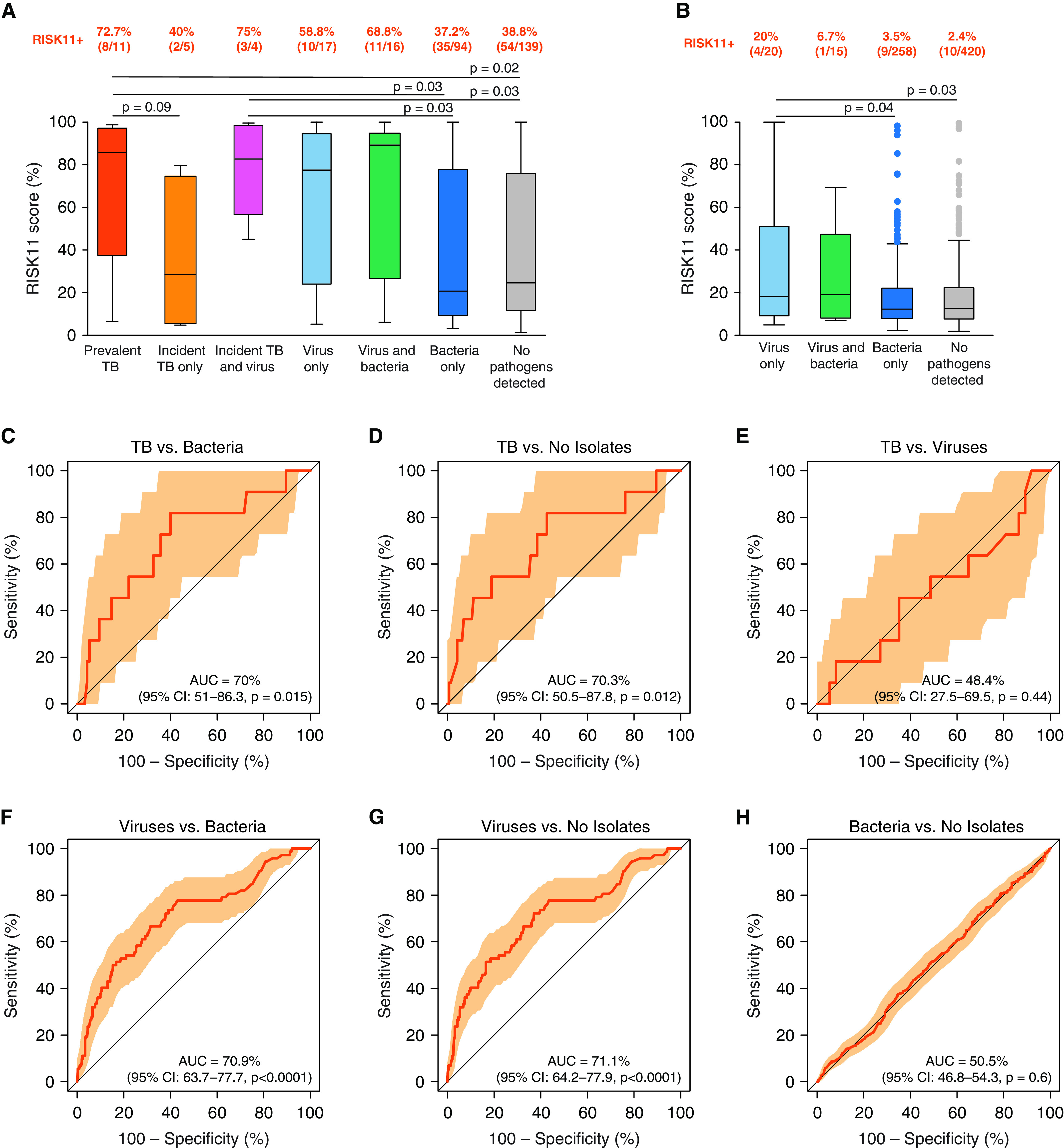
RISK11 performance for differentiating between participants with tuberculosis (TB), viral or bacterial upper respiratory tract organisms, and uninfected individuals. (*A* and *B*) Distributions of RISK11 scores in participants investigated for TB (*n* = 286) (*A*) and participants not investigated for TB (*n* = 713) (*B*). Only *P* values below 0.1 are shown. Boxes represent the interquartile range, and horizontal lines represent medians. The whiskers represent the lowest and highest RISK11^+^ score within 1.5 times the interquartile range from the lower quantile and upper quantile, respectively. The proportions of RISK11^+^ participants at each time point are indicated above each plot. (*C–H*) Performance of RISK11 in differentiating between participants with TB and participants with a bacterial upper respiratory organism (*C*), between participants with TB and uninfected participants (*D*), between participants with TB and participants with a viral upper respiratory organism (*E*), between participants with a viral upper respiratory organism and participants with a bacterial upper respiratory organism (*F*), between participants with a viral upper respiratory organism and uninfected participants (*G*), and between participants with a bacterial organism and uninfected participants (*H*). Shaded areas represent the 95% confidence interval (CI). AUC = area under the curve.

Because IFN-stimulated gene pathways are upregulated in HIV viraemia, we asked if other infections were also associated with elevated RISK11 scores. In total, 1,000 participants (148 RISK11^+^, 851 RISK11^−^, and 1 RISK11 indeterminate) were enrolled into the respiratory organism cohort (Figure 1B and Table E3). Of these, 23 were coenrolled into the HIV-uninfected longitudinal cohort and 286 were coenrolled in CORTIS, which required prospective TB investigation (Figures 1B and E1). All 999 participants with RISK11 results were screened for upper respiratory tract organisms regardless of symptoms. Viral and/or bacterial organisms were detected in 7.2% (72/999) and 38.9% (389/999) of participants respectively, with viral–bacterial codetection in 3.2% (32/999) of participants. RISK11 scores were significantly higher (*P* < 0.001) in participants with any viral organism (median [IQR], 46.7% [16.7–93.9%]) or both viral and bacterial organisms (42.8% [13.0–89.2%]) compared with those with only bacterial (13.4% [8.1–29.0%]) or no organisms (14.2% [7.8–30.1%]) (Figures 4A and 4B and Table E4).

Only three participants (0.3%) in the respiratory organism cohort presented with symptoms consistent with TB disease, including night sweats (0.3%, 3/999), cough (0.2%, 2/999), and loss of weight (0.2%, 2/999). RISK11 scores were higher (*P* = 0.02, Wilcoxon rank-sum test) in those with TB symptoms (median, 65.4%; IQR, 45–98.3%) compared with 996 participants without TB symptoms (median, 14.6%; IQR, 7.9–32.6%).

Fifty (5%) participants reported flu-like symptoms, of whom upper respiratory tract viruses or bacteria were detected in 8 (16%) and 24 (48%) participants, respectively. RISK11 scores were not different (*P* = 0.14) between participants with (median [IQR], 20.5% [8.7–47.5]) and without flu-like symptoms (*n* = 949; median [IQR], 14.5% [7.9–32.5]).

In total, 286 (28.6%) participants were coenrolled into CORTIS and investigated for TB at enrollment (Figure E1), of whom 11 (3.8%) had prevalent TB and 9 (9/275) later developed incident TB (Figure 1B). Baseline RISK11 scores were higher (*P* = 0.04) in participants with prevalent TB (median [IQR], 85.7% [37.2–97.4%]) compared with controls (26.6.% [11.7–84.9%]), but not (*P* = 0.46) in participants with incident TB (67.5% [28.6–79.6%]) compared with controls. Eight (72.7%) of the 11 participants with prevalent TB and 5 (66.7%) of the 9 participants with incident TB were RISK11^+^. Upper respiratory tract bacteria were detected at baseline in four (36.4%, 4/11) prevalent and two (22.2%, 2/9) incident TB cases, respectively.

No viruses were detected in participants with prevalent TB. However, the proportion of individuals with a viral organism was significantly higher (*P* = 0.02) in those who progressed to incident TB (44.4%, 4/9) than in those who remained healthy (12.4%, 33/266). Participants with a viral organism were five times more likely to progress to pulmonary TB than those without a viral organism (incident rate ratio, 4.99; 95% CI, 0.99–23.17).

### Organisms Associated with RISK11^+^ Status

Next, we sought to investigate the role and effect magnitude of individual viruses on RISK11 score. Multiple linear regression was performed in the 286 participants investigated for both upper respiratory tract organisms and TB to predict RISK11 scores based on detection of influenza, rhinoviruses, or coronaviruses, while adjusting for TB (Table 1). Residuals from the model were normally distributed (Figure E4). These variables significantly predicted RISK11 score (model *P* = 0.001); however, the adjusted *R*^2^ suggests that this model accounts for only 6% variation in RISK11 score. The three organisms independently predicted RISK11 score after accounting for interaction (*P* < 0.05). The odds (95% CI) of being RISK11^+^ were 4.0 (1.5–10.6), 39.6 (30.6–51.1), and 2.4 (1.0–5.5) times higher in participants detected with coronaviruses, influenza, and rhinoviruses, respectively, independent of TB risk, compared with those in whom such viruses were not detected. An exploratory multiple linear regression model in participants not investigated for TB showed similarly that the presence of viral rhinoviruses was significantly associated with higher odds of RISK11-positivity (Table E5).

**Table 1.** Univariable and Multivariable Linear Regression Models for Sociodemographic and Microbiologic Factors Associated with RISK11 Score in the Respiratory Organisms Cohort

Variable	*N* = 286	Univariable Analysis		Multivariable Analysis		
		**β** Coefficient (95% CI)	*P* Value	**β** Coefficient (95% CI)	Odds Ratio (95% CI)	*P* Value
Age, yr, median (IQR)	27 (22 to 34)	0.01 (−0.01 to 0.02)	0.266	—	—	—
BMI, kg/m^2^, median (IQR)	22.5 (19.1 to 28)	0 (−0.02 to 0.02)	0.969	—	—	—
Sex, M, *n* (%)	116 (40.6)	−0.24 (−0.54 to 0.05)	0.108	—	—	—
Adenovirus, *n* (%)	1 (0.4)	4.45 (4.3 to 4.59)	<0.001	—	—	—
Coronavirus (NL63, 229E, OC43, and HKU1), *n* (%)	10 (3.5)	1.3 (0.32 to 2.28)	0.01	1.38 (0.39 to 2.36)	4.0 (1.5 to 10.6)	0.006
Influenza (A, B, C, and H1N1), *n* (%)	3 (1.1)	3.57 (3.31 to 3.82)	<0.001	3.68 (3.42 to 3.93)	39.6 (30.6 to 51.1)	<0.001
Rhinoviruses, *n* (%)	21 (7.3)	0.8 (−0.03 to 1.64)	0.052	0.88 (0.04 to 1.71)	2.4 (1 to 5.5)	0.039
Prevalent tuberculosis, *n* (%)	11 (3.9)	0.97 (−0.49 to 2.42)	0.192	1.06 (−0.4 to 2.52)	2.9 (0.7 to 12.5)	0.154
Incident tuberculosis, *n* (%)	9 (3.1)	0.41 (−0.9 to 1.72)	0.541	—	—	—
*Haemophilus influenzae*, *n* (%)	65 (22.7)	−0.04 (−0.38 to 0.3)	0.808	—	—	—
*Klebsiella pneumoniae*, *n* (%)	8 (2.8)	−0.57 (−1.19 to 0.06)	0.075	—	—	—
*Moraxella catarrhalis*, *n* (%)	18 (6.3)	0.46 (−0.07 to 0.98)	0.091	—	—	—
Parainfluenza (types 1–4), *n* (%)	1 (0.4)	4.92 (4.77 to 5.06)	<0.001	—	—	—
Respiratory syncytial virus (A or B), *n* (%)	2 (0.7)	0.57 (−0.25 to 1.4)	0.171	—	—	—
*Staphylococcus aureus*, *n* (%)	28 (9.8)	−0.05 (−0.59 to 0.49)	0.865	—	—	—
*Streptococcus pneumoniae*, *n* (%)	32 (11.2)	−0.22 (−0.65 to 0.22)	0.333	—	—	—

*Definition of abbreviations*: BMI = body mass index; CI = confidence interval; IQR = interquartile range.

Odds ratios were obtained by exponentiating the β coefficients and are only shown for variables in the multivariable model.

### RISK11 Cannot Differentiate between Prevalent TB and Presence of Upper Respiratory Tract Viral Organisms

Among 286 participants investigated for TB and upper respiratory tract organisms, RISK11 differentiated prevalent TB (*n* = 11) from bacterial (*n* = 94, AUC, 70.0%; 95% CI, 51.0–86.3%) or no organisms (*n* = 139, 70.3%; 95% CI, 50.5–87.8%) with moderate performance (Figures 4C and 4D). However, RISK11 could not distinguish between prevalent TB and viral organisms (*n* = 37, AUC, 48.4%; 95% CI, 27.5–69.5%) (Figure 4E).

Among 988 participants without prevalent TB, RISK11 discriminated between 68 participants with viral organisms and the 352 participants with only bacterial organisms (AUC, 70.9%; 95% CI, 63.7–77.7%) (Figure 4F), and the 559 participants without organisms (AUC, 71.1%; 95% CI, 64.2.–77.9%) (Figure 4G). RISK11 did not discern between participants with bacteria only and those with no organisms (AUC, 50.5%; 95% CI, 46.8–54.3%) (Figure 4H).

## Discussion

We evaluated longitudinal kinetics of the RISK11 host blood transcriptomic signature of TB risk in HIV-uninfected and HIV-infected individuals, with and without TPT. Our results suggest that sustained RISK11 positivity is rare. Rather, positive RISK11 status is transient in people not treated with TPT, reverting to negative within 3 months in the majority of HIV-uninfected individuals and a large proportion of PLHIV. Those receiving TPT displayed the same kinetics, suggesting that TPT was not the primary mediator of RISK11 reversion. Our data suggest that intercurrent respiratory viral infections, such as rhinovirus and coronavirus, drive signature conversion and transient positive TB risk status. It is also clear that HIV infection elevates RISK11 signature scores, as reported previously (12, 17). A recent study of healthy TB-exposed individuals living in a TB-endemic setting demonstrated that the majority had detectable *Mtb* DNA in peripheral blood leukocytes (18). Detectable *Mtb* DNA was not associated with IFN-γ release assay positivity, but significantly decreased after isoniazid preventive therapy. These findings suggest that undiagnosed, subclinical *Mtb* infection may contribute to transient RISK11^+^ results observed in our study. RISK11 positivity was more frequent in PLHIV than those without HIV; RISK11 scores were higher in ART-naive than ART-experienced PLHIV; and RISK11 scores fell after ART initiation. Despite these findings, we note that RISK11 had good diagnostic performance for symptomatic TB and excellent prognostic performance for incident TB within 6 months of testing in HIV-uninfected (13) and HIV-infected people (17).

Nevertheless, we infer that upper respiratory and other systemic viral infections contributed to the RISK11^+^ prevalence rate in the CORTIS (9.3%) and CORTIS-HR (34.8%) study populations (13, 16, 17). Since TPT had a negligible effect on RISK11 status, it appears that RISK11 positivity due to active *Mtb* infection is relatively uncommon, consistent with the much lower incidence of TB disease compared with RISK11^+^ prevalence. Our results highlight the important effects of upper respiratory and systemic viral infections on ISG-based transcriptomic signatures, as suggested previously (19–21). This illustrates the need for confirmatory testing and/or combined TB and viral screening, which is being implemented in TB-endemic settings for coronavirus disease (COVID-19). Alternatively, further development and validation of signatures, such as those proposed by Singhania and colleagues and Esmail and colleagues (15, 19), which are not markedly influenced by ISG modulation, is required.

A key question is whether upper respiratory viral infections not only affect transcriptomic signatures independent of TB risk, due to induction of type I IFN, but whether they also induce changes in immune control of *Mtb* infection and precipitate progression to TB disease (22). For example, we have shown that HIV infection increases ISG-based signature score in healthy controls, independent of TB, in addition to the known elevated risk for progression to TB in PLHIV. We present preliminary evidence of a possible association between respiratory viral organisms and risk of incident TB, supporting the hypothesis that respiratory viral infections may trigger TB disease progression, or that immune dysfunction increases susceptibility to both viral and *Mtb* infection. Participants with respiratory viral organisms had fivefold increased risk of TB progression compared with those in whom no viral organisms were detected (Table E6). Although based on small numbers, which limits the significance of the result, the intriguing finding that viral coinfection may be associated with increased risk of TB should be tested more rigorously in future studies. Previous studies have reported that viral respiratory coinfections are associated with accelerated progression or more severe TB disease (23, 24). Patients with TB with nasopharyngeal viral–bacterial coinfection were also likely to have more severe TB disease (25), while murine influenza infection led to impaired control of *Mtb* infection via a type I IFN-dependent mechanism (26). However, no association was found between upper respiratory organisms and pulmonary TB in a Cape Town pediatric cohort (27).

Our study had several limitations. First, our study cohorts were small and included too few incident TB cases to allow robust assessment of longitudinal signature dynamics in relation to progression to TB disease. Second, only 29% of participants in the respiratory organisms cohort provided sputum for TB investigation, and therefore TB disease could not be excluded in the remainder. We also did not seek alternate diagnoses in symptomatic participants without detected respiratory tract organisms or TB disease, nor did we seek to identify lower respiratory or gastrointestinal organisms, which may also modulate RISK11 scores. We therefore hypothesize that the remainder of the variation in RISK11 score that is not explained by the model may be explained by organisms and host factors that we did not measure. Finally, we acknowledge that a number of the respiratory organisms we tested for are not typically pathogenic and occur as commensals.

Several characteristics of the study design strengthen our results. First, participants were enrolled consecutively as they presented for screening and enrollment into the parent studies, thereby minimizing selection bias. Second, this study used both oropharyngeal and nasopharyngeal samples, which increases the detection rate of organisms. Third, to our knowledge this is the first study to investigate temporal dynamics of a transcriptomic signature of TB risk in HIV-uninfected and HIV-infected cohorts, with and without TPT, and to investigate perturbation of an ISG-based signature by common respiratory viral organisms in individuals at risk for incident TB disease.

### Conclusions

This study provides insight into the association between common viral coinfections and one of several published TB signatures (5, 6, 28), highlighting challenges for implementation of these biomarkers as new tools for TB control. Control for confounding factors associated with elevated host blood transcriptomic signature scores, including viral infection, may be critical for implementation as potential TB biomarker tests. It is not yet known to what degree these results are generalizable to other host blood TB transcriptomic signatures, a question that needs to be addressed.
